# The Childhood Cancer Data Initiative: Using the Power of Data to Learn From and Improve Outcomes for Every Child and Young Adult With Pediatric Cancer

**DOI:** 10.1200/JCO.22.02208

**Published:** 2023-06-02

**Authors:** Joseph A. Flores-Toro, Subhashini Jagu, Gregory T. Armstrong, David F. Arons, Gregory J. Aune, Stephen J. Chanock, Douglas S. Hawkins, Allison Heath, Lee J. Helman, Katherine A. Janeway, Jason E. Levine, Ellyn Miller, Lynne Penberthy, Charles W. M. Roberts, Eve R. Shalley, Jack F. Shern, Malcolm A. Smith, Louis M. Staudt, Samuel L. Volchenboum, Jinghui Zhang, Jean Claude Zenklusen, Douglas R. Lowy, Norman E. Sharpless, Jaime M. Guidry Auvil, Anthony R. Kerlavage, Brigitte C. Widemann, Gregory H. Reaman, Warren A. Kibbe, James H. Doroshow

**Affiliations:** ^1^ National Cancer Institute, Bethesda, MD; ^2^ St Jude Children's Research Hospital, Memphis, TN; ^3^ National Brain Tumor Society, Boston, MA; ^4^ University of Texas, San Antonio, San Antonio, TX; ^5^ Seattle Children's Hospital, Seattle, WA; ^6^ Children's Hospital of Philadelphia, Philadelphia, PA; ^7^ Osteosarcoma Institute, Dallas, TX; ^8^ Dana Farber Cancer Institute, Boston, MA; ^9^ Smashing Walnuts Foundation, Leesburg, VA; ^10^ Essex Management, Rockville, MD; ^11^ University of Chicago, Chicago, IL; ^12^ US Food and Drug Administration, Silver Spring, MD; ^13^ Duke University, Durham, NC

## Abstract

Data-driven basic, translational, and clinical research has resulted in improved outcomes for children, adolescents, and young adults (AYAs) with pediatric cancers. However, challenges in sharing data between institutions, particularly in research, prevent addressing substantial unmet needs in children and AYA patients diagnosed with certain pediatric cancers. Systematically collecting and sharing data from every child and AYA can enable greater understanding of pediatric cancers, improve survivorship, and accelerate development of new and more effective therapies. To accomplish this goal, the Childhood Cancer Data Initiative (CCDI) was launched in 2019 at the National Cancer Institute. CCDI is a collaborative community endeavor supported by a 10-year, $50-million (in US dollars) annual federal investment. CCDI aims to learn from every patient diagnosed with a pediatric cancer by designing and building a data ecosystem that facilitates data collection, sharing, and analysis for researchers, clinicians, and patients across the cancer community. For example, CCDI's Molecular Characterization Initiative provides comprehensive clinical molecular characterization for children and AYAs with newly diagnosed cancers. Through these efforts, the CCDI strives to provide clinical benefit to patients and improvements in diagnosis and care through data-focused research support and to build expandable, sustainable data resources and workflows to advance research well past the planned 10 years of the initiative. Importantly, if CCDI demonstrates the success of this model for pediatric cancers, similar approaches can be applied to adults, transforming both clinical research and treatment to improve outcomes for all patients with cancer.

## Teaser

Childhood Cancer Data Initiative is a national commitment to harnessing data in ways that accelerate childhood cancer research.

## INTRODUCTION

Childhood cancers represent approximately 1% of all newly diagnosed cancer cases and are the leading cause of disease-related death in children in the United States,^1,2^ with survivors often experiencing long-term adverse effects from the disease and/or its treatment.^3,4^ Encouragingly, 5-year pediatric cancer survival rates have increased over the past 50 years, improving from 58% in 1975^5^ to an estimated 85% in 2022.^6^ This increase, driven primarily by improved outcomes for pediatric hematologic malignancies, has unfortunately not been observed for all pediatric cancers, notably in central nervous system tumors, which collectively saw 5-year survival rates increase from 57% to 70% by 1995,^5^ but subsequently stall with only 74% estimated in 2022,^5,6^ while others, such as diffuse intrinsic pontine glioma, still remain below 3% 5-year survival rate.^7^ Furthermore, for some very rare pediatric malignancies, natural history is not fully understood, there are no standard treatments, and conducting clinical trials is difficult because of small patient numbers.^8,9^ These limitations, combined with the comparatively low incidence of pediatric compared with adult cancers,^1,2^ underscore the necessity to collect, aggregate, and make accessible the research and clinical data that could enable a better understanding of the drivers of these cancers.

**CONTEXT**

**Key Objective**
How can the National Cancer Institute (NCI) build a pediatric cancer data ecosystem as a resource for the entire care and research community?
**Knowledge Generated**
The NCI-led Childhood Cancer Data Initiative (CCDI) represents a novel approach to collecting, aggregating, and sharing data that can drive research to greatly accelerate treatment development and improve outcomes for patients with pediatric cancer, survivors, and their families. Through these efforts, the NCI will use CCDI as an exemplar to meet Moonshot goals for building its portion of a learning health care system for cancer to be used as a resource for the entire research and care community to more easily access and use critical data sets and tools needed for research.
**Relevance *(S. Bhatia)***
CCDI will serve a resource for the entire research and clinical care community for access and utilization of critical data sets and tools and serve as an exemplar to meet Moonshot goals.*
  *Relevance section written by *JCO* Associate Editor Smita Bhatia, MD, MPH, FASCO.

The Childhood Cancer Data Initiative (CCDI),^10^ announced during the 2019 President's State of the Union address,^11^ is a 10-year, $50-million (in US dollars)–per-year, federal investment to advance pediatric cancer research through both new data generation and development of platforms for sharing data. The vision of the initiative is to learn from every child, adolescent, and young adult (AYA, under CCDI, the patient group extending to age 39 years diagnosed with a pediatric cancer) by bringing together the research, advocacy, and care communities in an ambitious effort to build a Data Ecosystem^12^ that enables rapid and systematic data collection, sharing, access, and analysis. This will connect researchers with data and software tools from previously separate sources to allow integrated data analysis from multiple academic, community-driven, and National Cancer Institute (NCI)–supported data resources. These efforts are meant to accelerate progress in etiology, prevention, diagnosis, treatment, outcomes, quality of life, and survivorship discoveries for pediatric and AYA patients with cancer.

Although initiated by NCI, the CCDI is designed to be a collaborative community endeavor with support for new and ongoing community efforts, as well as be a springboard for other critical work needed to extract the greatest benefit from the available data. To that end, the institute is working across the entire pediatric cancer community, including patients, providers, survivors, families/caregivers, advocates, clinical and basic researchers, foundations, and industry. By engaging broadly, we will ensure the needs of all stakeholders inform the goals of the initiative. Within its role, the NCI will aim to provide data resources designed to evolve and grow in scope and utility, well beyond the planned 10 years of the CCDI.

In June 2020, a multidisciplinary working group, convened by the NCI's Board of Scientific Advisors (BSA), generated a report^13^ to guide the CCDI. The report summarizes overarching goals, including collecting, analyzing, and sharing all relevant data and building processes to transform those data into knowledge. The working group detailed 24 specific recommendations to advance discovery in meaningful ways, broadly grouped into two main categories.

- Aggregate and generate broad categories of data to enable improved treatments and outcomes, from diagnosis through survivorship
- Develop infrastructure to support a comprehensive data ecosystem

**FIG 1.**
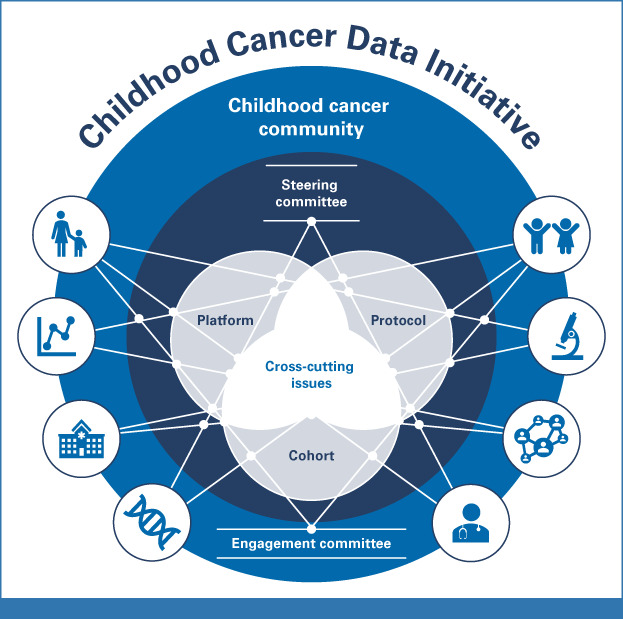
CCDI Working Group structure: The CCDI Working Groups provide important feedback on the crucial areas on which the initiative should focus. The Cohort Working Group is tasked with developing strategies and a series of initiatives (eg, capture historical data and ensure it is accessible and interoperable with the prospective data) to gather data from every patient diagnosed with a pediatric cancer in the United States. The Protocol Working Group provides expert guidance for the MCI screening protocol. The Cross-Cutting Working Group is focused on ensuring the effective collaboration, communication, and coordination of all activities for the CCDI. The Platform Working Group defines a strategy and approach for federating and/or integrating data to enable integrated analytics and incentivize researchers to use the data in new ways. CCDI, Childhood Cancer Data Initiative; MCI, Molecular Characterization Initiative.

The CCDI presents an opportunity to develop a unique programmatic structure within the cancer research landscape. Recent programs such as Cancer Moonshot,^14^ Gabriella Miller Kids First Pediatric Research Program (Kids First),^15^ Pediatric Molecular Analysis for Therapy Choice (Pediatric MATCH),^16^ My Pediatric and Adult Rare Tumor network (MyPART),^17^ and Therapeutically Applicable Research to Generate Effective Treatments (TARGET),^18^ among others, have laid the foundation for the functioning of CCDI's data collection and Childhood Cancer Data Ecosystem.^12^ However, the CCDI will explore ways to expand to accommodate new programs. To achieve this, we invited a diverse array of experts from across the childhood cancer community to participate in focused working groups to help guide the vision of the CCDI. These expert working groups (Fig 1) focus on cohort building, data infrastructure development, molecular characterization, cross-cutting issues, and community engagement.

Composed of researchers, data scientists, clinical oncologists, and patient advocates, each group ensures CCDI priorities align with the needs of the entire community to guide and assess the direction of the initiative. As membership includes both internal (NCI) and external partners, these working groups, along with the steering committee (Fig 1), provide oversight for the initiative. CCDI will be regularly evaluated using metrics including bibliometric analysis, usage of data, collaborative activities, and assessment of major scientific accomplishments. In addition, CCDI leadership regularly report to the NCI BSA/National Cancer Advisory Board (NCAB)^19^ on the progress of the initiative, and ultimately all activities will stem from this guidance.

## CCDI ACTIVITIES

### Aggregate and Generate Broad Categories of Data

Creating more comprehensive and meaningful data sets will help researchers and clinicians better understand the biology of pediatric cancers, develop new therapies, and assess long-term effects of cancer treatment. The traditional paradigm of data collection, or generation for a specific need, is shifting to include data use for novel discovery and hypothesis generation. Within this shift, CCDI is working to fill knowledge gaps by generating and augmenting key data sets and allowing for expanded analytics.

As an initial step toward this goal, the NCI initiated the Molecular Characterization Initiative (MCI)^20^ in collaboration with the Children's Oncology Group (COG)^21^ in 2022. The MCI offers free, clinically annotated, genomic characterization of tumors and germline samples to patients and their physician. This national strategy for molecular characterization exemplifies CCDI's commitment to generating and collecting new data to drive treatment development for pediatric cancers.

NCI contracted molecular characterization to Frederick National Laboratories for Cancer Research who, through a competitive review and selection process, subcontracted this work to the Steve and Cindy Rasmussen Institute for Genomic Medicine (IGM) of Nationwide Children's Hospital. IGM performs the genomic characterization (Table 1) and provides clinical reports to care providers and their patients within 2 weeks of testing. After characterization, research grade data are deposited to the NCI's Cancer Data Service^22^ and can be accessed through a controlled data access request to the database of Genotypes and Phenotypes (dbGaP, PHS002790^23^).

**TABLE 1.**
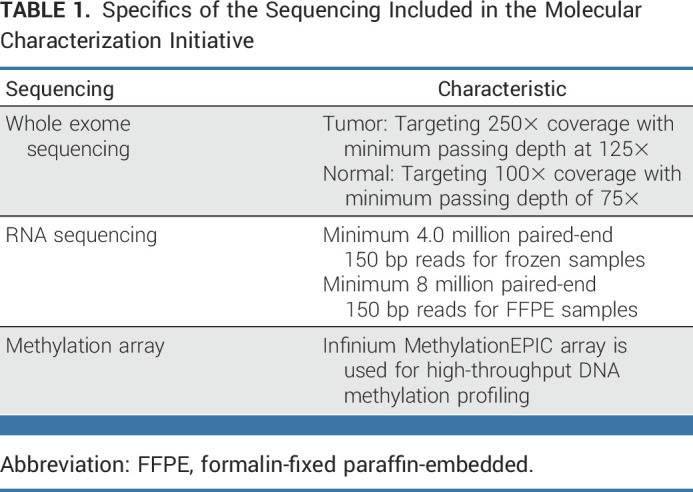
Specifics of the Sequencing Included in the Molecular Characterization Initiative

Implementing the MCI was a complex undertaking, involving intersections of patient care, pathology, tumor banking, and genomic assays. To develop and optimize the workflows, initial efforts focused on high-need areas, which included newly diagnosed central nervous system tumors, soft tissue sarcomas, and rare childhood cancers.^24^ Partnership with the COG's Project:EveryChild (PEC)^25^ enabled rapid rollout of the MCI on a national scale and patient consent allows NCI to collect clinical and genomic data on every patient enrolled in PEC. The MCI provides value to the treating physician, the patient, and the family by returning a diagnostic molecular profile that may help refine diagnosis and treatment plans,^26,27^ as well as inform clinical trial options and the need for genetic counseling. At the same time, the broader pediatric research community benefits from consistent validated molecular data that are linked to the clinical data. These standardized molecular data will enable hypothesis testing across multiple cancer types and histologies and allow researchers to posit new questions. Through the first year of the initiative, 751 participants have been enrolled and characterized, and the molecular characterization data from 630 have been made available.

As the MCI progresses, the CCDI will assess opportunities for expansion, including

- Developing mechanisms to expand availability of MCI to patients currently treated outside of COG institutions
- Providing special emphasis on access to the MCI for historically marginalized communities
- Expansion to additional patient populations, including those with relapsed/refractory cancers
- Addition of disease-specific molecular assays using banked specimens linked to clinical data, including treatment outcomes
- Developing mechanisms to assure longitudinal follow-up to facilitate cohort studies investigating toxicities and survivorship

In addition, the MCI team is evaluating opportunities to improve the usefulness of returned data, which could include more detailed reports or assisting with clinical trial matching. The group is also exploring how data could be shared across organizations, so decisions by institutional tumor boards, or outcomes of novel treatments, could prospectively inform future treatment choices and generate the scientific rationale for novel clinical investigations.

The CCDI is enabling further efforts to enhance our understanding of the biology of certain pediatric cancers. This approach includes strategic investments in germline and primary tumor characterization of multiple tumor types and aims to eventually include secondary cancers and metabolomic profiling of children with inherited predisposition to cancer. Extending from this, the CCDI has supported the characterization and development of pediatric preclinical models such as cell lines, patient-derived xenografts, and solid tumor organoids as a part of the NCI Pediatric Preclinical in Vivo Testing (PIVOT)^28^ program. These models are integral to drug development and will improve the speed and efficacy of translational medicine in pediatric cancer. The CCDI has also supplemented funding for research gene sequencing of samples from the Pediatric MATCH, Childhood Cancer Survivor Study, and the Children's Brain Tumor Network. Subsequently, CCDI initiated plans to develop a Coordinated National Initiative on Rare Pediatric Cancers as there is an unmet need to collect detailed registry data on very rare childhood cancers to better understand their natural history and accelerate progress in defining treatment of these cancers.

Further efforts to support inclusion of broad data sets include a focus on reducing health disparities for patients with pediatric cancer. Using data available from the National Childhood Cancer Registry (NCCR),^29^ the CCDI will focus on identifying gaps in outcomes of historically underserved populations. To enable this, experts in health disparities and social determinants of health are providing crucial input to help ensure all patients have access to CCDI resources, such as the MCI. These collective efforts will support and enhance ongoing NCI and NCI-supported pediatric cancer research, adding to the rich data sets that will be available through CCDI.

### Develop Infrastructure

Emerging collaborative data science platforms are poised to accelerate transdisciplinary discovery and clinical translation for pediatric cancers lacking effective standards of care. To achieve the goal of aggregating pediatric cancer data, CCDI is developing a Data Ecosystem.^12^ This network will comprise tools to bring together formerly disconnected clinical, research, and registry data on children and AYAs. Users will have easy access to the ecosystem through a centralized hub, which enables the discoverability and sharing of clinical and research data. The ecosystem will incorporate and harmonize preclinical, clinical phenotype and demographics, biological research, electronic health record (EHR), and registry data, and be extensible so that it can handle future data types and enable temporal and longitudinal queries. Representing the full patient trajectory, from initial diagnosis through treatment into survivorship, and improving the linkage of clinical annotations associated with generated molecular data are important goals for the data ecosystem.

To further enable the ecosystem, CCDI has developed a data coordination center to improve data submission, access, and interoperability. In this structure, CCDI curators will be available to help submitters with data QC, standardization, and the harmonization process. Standard driven data model templates to collect metadata and clinical data were created to capture participant-level clinical data that facilitates interoperability between -omics data (genomic or variant) and the EHR.

Interacting with data in different repositories can be cumbersome and confusing. A key objective of CCDI is to make data more useful by connecting data from different sources using a federated model, allowing multiple databases to function as one through a centralized portal. This portal will enable exploration, integration, and collaboration across childhood cancer data resources (eg, Kids First Data Resource Center,^30^ St Jude Cloud,^31^ UCSC Treehouse,^32^ Pediatric Cancer Data Commons [PCDC],^33^ NCI Cancer Research Data Commons [CRDC],^34^ and the NCCR). Enabling interoperability requires community standards such as Global Alliance for Genomics and Health,^35^ Fast Healthcare Interoperability Resources (FHIR),^36^ minimal Common Oncology Data Elements (mCODE),^37^ and the NCI Thesaurus (NCIt)^38^ to be used and extended. It also requires implementing common application programming interfaces, standard terminologies, and harmonized common data elements. Additionally, through development of tools, methods, and pipelines that package, clean, or analyze data, CCDI is working to improve the quality and consistency of clinical and research data to maximize its utility.

**FIG 2.**
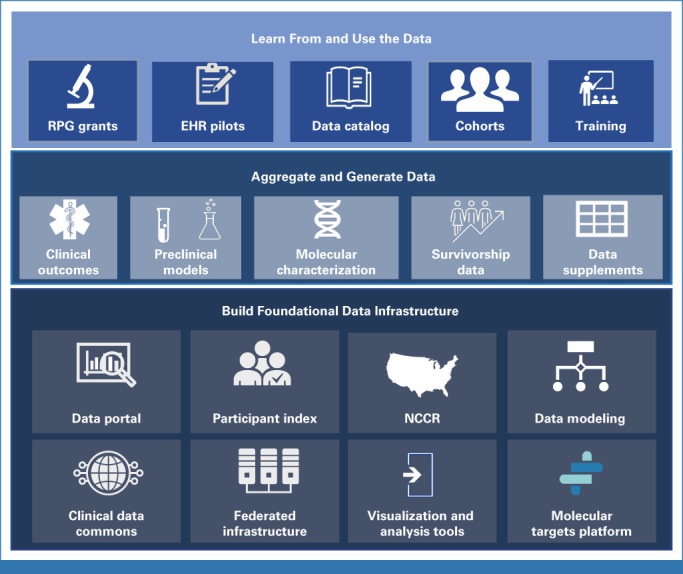
CCDI activities over the first 3 years: Broad categorical breakdown of the first 3 years of CCDI activities. Roughly equal funding contributions have been made to each of these objectives over the first 3 years. CCDI, Childhood Cancer Data Initiative; EHR, electronic health record; NCCR, National Childhood Cancer Registry; RPG, research project grant.

While the CCDI Ecosystem is under development, a range of tools and resources will be made available and connected to make it easier to locate and use data. To date, several of these have already been made available through CCDI (Fig 2).

- The CCDI NCCR is a rapidly growing public data repository on childhood and AYA cancer incidence, outcome data, and statistics. NCCR intends to connect data from myriad sources, such as pharmacy, cancer registry, and administrative data from claims as well as hospital EHRs, cancer research centers, genomic testing, and others. Future data products for the NCCR include residential history, social determinants of health, longitudinal treatments, and outcomes data including recurrence and subsequent cancers. In addition to the broad efforts to fully enumerate and characterize children diagnosed annually, and the development of a prospective cohort for molecular characterization, the NCCR will assure long-term follow-up of a prospective cohort for evaluation of late mortality, cancer recurrence, and subsequent malignant neoplasms.
- The CCDI Childhood Cancer Data Catalog is an inventory of childhood cancer data resources and tools. This catalog makes it easier to find disparate data that may help answer important research or clinical questions. Each resource page includes a summary description, data content types, and links to access the data. The inventory includes childhood cancer repositories, registries, data commons, websites, tools, and catalogs that manage and refer to data.
- The CCDI Molecular Targets Platform (MTP)^39^ is a knowledgebase that allows researchers to browse and identify associations between molecular targets, diseases, and drugs specific for childhood cancers. The tool extracts, organizes, annotates, and links to data from multiple sources. This can be used for systematic identification and prioritization of the Food and Drug Administration's Relevant Molecular Target List (MTP).^40^ The MTP allows comparisons of childhood datasets with tabular and graphic visualizations of genomic data and facilitates prioritization of targets by reducing the time spent monitoring and organizing scientific and medical literature.

In addition to these already launched applications, additional resources are in preparation, and are anticipated for release in 2023.

- NCI is committed to ensuring CCDI data will be easily findable and accessible. To accomplish this, CCDI is developing a hub that will serve as an entry point to access all CCDI resources (eg, data, tools, web applications, etc). This will also include a user interface to query for data assets.
- The CCDI data ecosystem comprises retrospective and prospective data on participants that have been collected under multiple protocols and at multiple sites, resulting in data for the same participant being attached to multiple IDs. For CCDI to be effective, it is critical to cross-reference data. So, a participant index that collects all known research IDs and creates a mapping table enabling integrated analysis of data is being developed.

The CCDI has also funded (through grant supplements and contract mechanisms) multiple efforts to develop and refine computational methods, analytic and management tools, and pipelines to be shared with or incorporated into NCI resources for use with a variety of clinical and research data sets.

- Pilot efforts are exploring the feasibility of automating the abstraction of clinical data from patient medical records to integrate discrete patient data into the EHR. Furthermore, Pediatric Early Phase Clinical Trial Network in collaboration with the Pediatric Brain Tumor Consortium piloted a study to enable electronic laboratory data transfer from participating institutions to MediData/RAVE. These efforts aim to learn about adverse events and outcomes from the treatment, payment, and operations of health care activities from state public health authorities to maximize the impact of these data.
- Pilot studies are also underway to address critical issues such as quality assurance and data harmonization, and featuring pediatric cancer variant content in the Clinical Interpretations of Variants in Cancer^41^ knowledgebase.
- Work is in progress on automated curation of data (eg, natural language processing) for refining, scaling, and capture of real-world data. These funded projects include functional capabilities that address multiple areas, including better interpretation of pathology images and patient reports.
- The pediatric Proton/Photon Consortium Registry's data on disease, treatment, and clinical outcomes of patients with pediatric cancer receiving radiation therapy will be part of the NCCR, and focus on investigating the risks and benefits of pediatric proton/photon therapy and genetic susceptibility to adverse radiation-treatment effects. This could potentially expedite radiation outcomes-based research. The activities completed include development of radiation specific standard common data elements and harmonization.
- A rare pediatric tumor atlas using single-cell analyses and spatial transcriptomics along with computational methods to study tumor heterogeneity and genetic interactions is being developed. Associated clinical data, patient reported outcomes, and biospecimen data (whole exome sequencing, RNA seq, methylation, and TruSight Oncology 500 gene panel sequencing) will be collected and deposited.

The CCDI aims to incorporate all relevant information and data sources, to test and refine systems and workflows, and to develop a data ecosystem capable of accomplishing its aggressive goals. The key components of the data platform will increase the availability, usability, research value, and translational impact of a broad collection of childhood cancer data. Collecting data along all points of a patient's cancer trajectory will enable research and clinical communities to learn from every child with cancer. In this way, the CCDI Data Ecosystem will facilitate much-needed progress for treating pediatric cancers.

## ENSURING DIVERSE REPRESENTATION AND EMPOWERING PATIENTS

The CCDI is committed to ensuring that it remains responsive to the needs of the entire community. As such, it is critical to leverage clinical and demographic data from all children with cancer, including historically under-represented groups, patients treated at smaller rural or community-based institutions, and AYA patients diagnosed with pediatric cancers, all of whom have poorer health outcomes.^42,43^ We are working broadly with the pediatric and AYA community to better understand the barriers to participating in CCDI activities.

AYA patients with cancer, defined by the NCI as being diagnosed with cancer between age 15 and 39 years, face many challenges. To this end, CCDI will include AYA patients with a cancer primarily diagnosed in children, allowing for study of family history, disease course, and outcomes across a broad range. However, the Coordinated National Initiative on Rare Pediatric Cancers will consider any rare cancer of unmet medical need occurring in children and AYAs, including cancers that primarily occur in adults. A prime function of the CCDI Engagement Committee is to offer guidance on engaging with all members of the community, including AYA patients. We have begun identifying clinical investigators with a special interest in AYA populations, as well as AYA advocacy groups and their representatives to be included in panels and workshops, ensuring those voices are heard.

Furthermore, overcoming geographic or socioeconomic barriers as well as assessing institutional barriers to patient enrollment or sharing of data or samples will be a focus for CCDI. This assessment will allow us to determine whether CCDI activities have an impact on improving health disparities. For instance, some sites may lack the requisite infrastructure to participate in the MCI, such as smaller community centers or regional facilities, while others may be operating outside of established relationships or with more specialized networks. Establishing and maintaining these partnerships between the CCDI and the broader community will help the CCDI properly guide and focus future activities.

In the rapidly evolving biomedical data field, ethically collecting deidentified data from patients requires they and their families are fully informed on how their data and tissues will be used. CCDI has engaged experts to help develop unified guidance on patient informed consent language, aiming to ensure the patient and family receive adequate information to make decisions and the data are collected in such a way as to allow for broad secondary research use. Those data are made available in open- and controlled-access tiers, as required by the informed consent, to protect patient privacy and confidentiality. To ensure data security, the NCI's Data Access Committee reviews data access requests for all controlled-access data. Furthermore, it is crucial to gain lifetime consent whenever possible, or permission to recontact the patient when a child becomes an adult, so that samples and data can be used for research over time. Planning is underway regarding the development of a portal to empower patients to contribute their own data directly to the CCDI.

## CROSS-CUTTING APPROACHES WITHIN THE CCDI

A central feature of the CCDI is its novel approach to data sharing. Building on the foundation of existing projects to assemble a data ecosystem capable of providing comprehensive clinical and genomic data on every patient with pediatric cancer is critical to the CCDI's mission. Issues of cross-cutting significance, such as data collection, standards, and governance, need to be addressed across CCDI activities in a consistent manner. To accomplish this, a cross-cutting team (including research and data scientists, clinical oncologists, patient advocates, etc) was created to facilitate transparent collaboration, communication, and coordination of common concerns across working groups and the research community and develop guidelines to address these needs. From these activities, pilot studies can be designed and implemented to test various aspects of cross-cutting significance, such as data standards and harmonization, universal participant identifiers, data governance, and patient consent. These initial projects, combined with lessons learned from solutions already in use by the community (eg, Pathology, Radiology/Imaging, Signs/Symptoms, Medical Oncologist assessment, and bioMarkers,^44,45^ FHIR, OncoTree,^46^ mCODE, Observational Medical Outcomes Partnership,^47^ NCIt, North American Association of Central Cancer Registries,^48^ and international standards developed by the PCDC^49,50^), will provide valuable support for the development of long-term strategies to address critical needs.

In addition to the central coordination of pilot project design and implementation, the cross-cutting team is working to facilitate communication with the pediatric cancer community. This includes programs such as NCI's CRDC,^34^ and external programs such as the National COVID Cohort Collaborative^51^ and the NIH Cloud Platform Interoperability effort.^52^ The CCDI is also examining the potential for collaborating with industry and international partners. Exploring these options will help the CCDI benefit from existing solutions, avoid mistakes, and learn from groups who have built innovative systems with similar aims. Engaging a wide set of stakeholders will help build long-term sustainability, ensuring the tools and ecosystems remain robust and useful far past the proposed 10-year life span of the initiative.

In conclusion, the CCDI is a collaborative, community-based initiative that will expand on existing projects and tools, while building a data ecosystem that will facilitate learning from every child and AYA with a pediatric cancer. Research and clinical care data are the foundations that will promote discovery to improve the lives of patients, survivors, and their families. The NCI has a unique ability to facilitate the broad sharing of data generated from publicly funded projects to support fundamental research and treatment for patients with pediatric cancer. The CCDI is committed to activities that could translate into impactful discoveries and safe and effective novel therapeutic options.

The success of a broad initiative like CCDI, which intersects many systems and financial structures, needs bidirectional input from all stakeholders and active oversight. A significant challenge for CCDI is the nature of funding, which will necessitate efficient planning to maintain progress while being dependent on the annual federal budget cycle. Furthermore, as the CCDI is heavily reliant on the community, coordinating efforts that span multiple independent administrative systems, external funding mechanisms, and management structures will require directional communication and management strategies to ensure all parties are able to efficiently work with each other and resolve potential conflicts of interest. Progress through the first 3 years of the initiative, discussed at the December 2022 meeting^53^ of the NCI's BSA/NCAB,^54^ provides assurance of the ability of CCDI to meet these challenges.

Bidirectional community engagement and collaboration with all stakeholders will ensure all perspectives are incorporated and activities are aligned with the needs of research and care. The CCDI activities highlighted here are expected to continue and expand over the remaining years of the initiative. By demonstrating the success of this model in pediatric cancers, similar approaches can be developed for adult cancer research and care, and thus aid in improving outcomes for all patients with cancer.

## APPENDIX 1. AUTHOR CONSORTIUM

### Collaborators

Richard Aplenc, MD, MSCE, PhD: Children's Hospital of Philadelphia, Philadelphia, PA; Caitlyn W. Barrett, PhD: CureSearch, Baltimore, MD; Naomi Bartley, MS: AstraZeneca, Gaithersburg, MD; Vicki Buenger, PhD: Coalition Against Childhood Cancer (CAC2), Philadelphia, PA; Ethan Cerami, PhD: Dana Farber Cancer Institute, Boston, MA; Susan L. Cohn, MD: University of Chicago, Chicago, IL; Susan I. Colace, MD: Nationwide Children's Hospital, Columbus, OH; Carrye R. Cost, MD: University of Colorado, Aurora, CO; Brian D. Crompton, MD: Dana Farber Cancer Institute, Boston, MA; Tanja M. Davidsen, PhD: National Cancer Institute, Bethesda, MD; Sharon J. Diskin, PhD: Children's Hospital of Philadelphia, Philadelphia, PA; Jamie Ennis Bloyd, MPA: American Childhood Cancer Organization, Lexington, KY; Suzanne J. Forrest, MD: Dana Farber Cancer Institute, Boston, MA; Maryam Fouladi, MD, MSc: Nationwide Children’s Hospital, Columbus, OH; Amar Gajjar, MD: St Jude Children's Research Hospital, Memphis, TN; Julia Glade Bender, MD: Memorial Sloan Kettering Cancer Center, New York, NY; Nancy Goodman, JD, MPP: Kids v Cancer, Washington, DC; Maria M. Gramatges, MD, PhD: Texas Children's Hospital, Houston, TX; Casey S. Greene, PhD: University of Colorado, Aurora, CO; Julie A. Guillot: Leukemia and Lymphoma Society, Rye Brook, NY; Amanda P. Haddock: Dragon Master Foundation, Kechi, KS; Melissa A. Haendel, PhD: University of Colorado, Aurora, CO; D. Ashley Hill, MD: Children's National, Washington, DC; Amie E. Hwang, MPH, PhD: University of Southern California, Los Angeles, CA; Daphne Kaas-Hogan, MD: Dana Farber Cancer Institute, Boston, MA; Edward A. Kolb, MD: Nemours Children's Hospital, Wilmington, DE; Andrew L. Kung, MD, PhD: Memorial Sloan Kettering Cancer Center, New York, NY; Salvatore La Rosa, PhD: Children's Tumor Foundation, New York, NY; Theodore W. Laetsch, MD: Children's Hospital of Philadelphia, Philadelphia, PA; Elizabeth R. Lawlor, MD, PhD: Seattle Children's Hospital, Seattle, WA; Philip J. Lupo, PhD: Baylor College of Medicine, Houston, TX; Troy A. McEachron, PhD: National Cancer Institute, Bethesda, MD; Clay McLeod, MS: St Jude Children's Research Hospital, Memphis, TN; Shannon K. McWeeney, PhD: Oregon Health and Science University, Portland, OR; Jill P. Mesirov, PhD: University of California, San Diego, San Diego, CA; Sarah Milberg, MPP: St Baldrick's Foundation, Monrovia, CA; Andy F. Olshan, PhD: University of North Carolina, Chapel Hill, NC; Corrie Painter, PhD: Broad Institute, Boston, MA; Donald W. Parsons, MD, PhD: Texas Children’s Hospital, Houston, TX; Aubrey Reichard-Eline: With Grace, Gillette, NJ; Karlyne M. Reilly, PhD: National Cancer Institute, Bethesda, MD; Adam C. Resnick, PhD: Children's Hospital of Philadelphia, Philadelphia, PA; Abby B. Sandler, PhD: National Cancer Institute, Bethesda, MD; Nita L. Seibel, MD: National Cancer Institute, Bethesda, MD; Nilay Shah, MD: Nationwide Children’s Hospital, Columbus, OH; David A. Siegel, MD, MPH: US Centers for Disease Control and Prevention, Atlanta, GA; Logan G. Spector, PhD: University of Minnesota, Minneapolis, MN; Alejandro Sweet-Cordero, MD: University of California, San Francisco, San Francisco, CA; Gail E. Tomlinson, MD, PhD: University of Texas, San Antonio, San Antonio, TX; Emily S. Tonorezos, MD, MPH: National Cancer Institute, Bethesda, MD; James V. Tricoli, PhD: National Cancer Institute, Bethesda, MD; Olena M. Vaske, PhD: University of California, Santa Cruz, Santa Cruz, CA; Mary Frances Wedekind Malone, DO: National Cancer Institute, Bethesda, MD; Brent R. Weil, MD: Boston Children's Hospital, Boston, MA; and Torunn Yock, MD: Massachusetts General Hospital, Boston, MA
